# Problematic social media use in 3D? Relationships between traditional social media use, social virtual reality (VR) use, and mental health

**DOI:** 10.1371/journal.pone.0314863

**Published:** 2025-01-15

**Authors:** Shay Xuejing Yao, Joomi Lee, Reed M. Reynolds, Morgan E. Ellithorpe

**Affiliations:** 1 Department of Communication, Georgia State University, Atlanta, GA, United States of America; 2 Department of Communication, University of Arkansas, Fayetteville, AR, United States of America; 3 Department of Communication, University of Massachusetts Boston, Boston, MA, United States of America; 4 Department of Communication, University of Delaware, Newark, DE, United States of America; University of Science and Technology of China, CHINA

## Abstract

This research expanded on prior work exploring the relationship between social media use, social support, and mental health by including the usage of social virtual reality (VR). In Study 1 (undergraduate students; n = 448) we examined divergent relationships between problematic social media use (e.g., Facebook, TikTok), total use, and users’ mental health indicators (e.g., depression, anxiety, social isolation). To determine whether problematic social media use patterns extended to immersive 3-D environments, we sampled active social VR users (e.g., Rec Room) in Study 2 (n = 464). Problematic social VR use was related to decreased real-life social support (β = -.62, 95%CI [-.80, -.44]), but not to VR social support (β = -.06, 95%CI [-.25, .14]). Conversely, the amount of social VR use was only related to increased social VR (β = .06, 95%CI [.04, .15]) but not to real-life social support (β = -.02, 95%CI [-.05, .04]). Study 2 also revealed a finding that may be unique to the 3-D immersive environment: the amount of social VR use facilitated better mental health for VR users, but only through stronger perceived social support on social VR but not in real life. This result highlights the potential of immersive media to promote mental well-being by facilitating engaging and meaningful social interactions.

## Introduction

Social media platforms have attracted billions of users worldwide, supporting users in building connections and networks [1, 2]. However, users may also be prone to problematic social media use [3, 4], characterized by symptoms resembling addiction, where users are preoccupied with social media and depend on it for mood management [5, 6]. Additionally, withdrawal, mood swings, and relapse may occur when users try to quit social media [7, 8]. Previous research on problematic social media use has largely focused on traditional 2-D social media applications (e.g., Tik Tok, Instagram), leaving a notable gap in our understanding of potential problematic media uses within immersive 3-D social virtual reality (VR) environments (e.g., VRChat, AltspaceVR). Although the technological affordances and applications of traditional social media and social VR differ, they both allow users to interact socially, build networks, and maintain relationships [9]. Given the rapidly increasing popularity of social VR [10], systematic investigation of the benefits and challenges of social VR use is becoming imperative. Through two studies, our research contributes to the current understanding of problematic social media use, particularly its association with perceived social support (e.g., in real life, online) and mental health (e.g., depression, anxiety, social isolation). Additionally, our research fills an important research gap by exploring whether these patterns apply to emerging VR social media platforms.

### Problematic social media use and mental health

A recent meta-analysis found significant correlations between problematic social media use and mental health, including depression, anxiety, and loneliness [11]. A systematic review revealed that problematic social media use and depression tend to be positively related, though the magnitude differs by gender identity [12]. Conversely, research on social media use that did not distinguish between problematic social media use and the more general amount of social media use found mixed results regarding the impact on mental health. Studies have found that increased time spent on social media may lead to greater depression [13] and diminished mental well-being [14]. Another study indicated that social media use contributes to greater loneliness and depression but also greater life satisfaction [15]. However, a longitudinal study found no evidence of direct effects between social media use and mental health (i.e., depression, anxiety) in adolescents [16].

These inconsistent findings might be due to the lack of differentiation between *problematic* and *amount of* social media use. There is one previous longitudinal survey that has tested the impact of both problematic social media use and the more general amount of social media use on depressive symptoms among teens and tweens [17]. It was found that problematic social media use, but not the amount of social media use, was associated with depressive symptoms one year and two years later. This study was informative about the possible different effects between problematic and the amount of social media use on mental health, however the target population was adolescents but not adults.

Regarding how to differentiate problematic social media use and the amount of social media use, some previous studies used an arbitrary cutoff number (e.g., five hours per day) to differentiate problematic and non-problematic social media use [18, 19]. However, problematic social media use may be influenced by factors other than time, such as alcohol use and social phobia [20]. For example, other research in the video gaming space has found that only about one third of heavy users (defined as playing 30+ hours per week) met the criteria for problematic use–indicating a large disconnect between amount of use and whether such use is problematic [21]. This means that longer hours on social media do not necessarily determine the type of use to be problematic. Here we define the amount of social media use as total time spent on social media per week and does not necessarily relate to addictive symptomatology.

Another important aspect of social media use is social support as a mediating mechanism between social media use and mental health. For instance, Lin, Namdar [22] found that perceived real-life social support mediated the association between problematic social media use and depression and anxiety. However, this research only tested the role of real-life but not online social support. Meshi and Ellithorpe [23] examined the mediating role of both real-life social support and social support on social media and found that only real-life social support, but not social support on social media, reduces the negative influence of problematic social media use on mental health. Specifically, this result indicated that problematic social media use was related to reduced perceived real-life social support but increased social media social support. Other research found a positive relationship between problematic Facebook use and social media social support [24]. However, it remains unclear whether the two types of social support (real-life and online) are differentially influenced by problematic social media use and the amount of social media use.

Social support is important because previous research finds that real-life social support is associated with reduced depression [25], anxiety [26], and social isolation [27]. The research on social media social support, however, is more equivocal. A systematic review found that Facebook-based social support reduces symptoms of mental illness [28], whereas other research has found social media social support linked to greater depression [29]. When considering real-life social support and social media social support together, research findings are inconsistent. Several studies have found that real-life social support, but not social media social support, contributes to decreased depression, anxiety, and social isolation, and increased life satisfaction [23, 30, 31]. Other research found that both real-life social support and social media social support reduce depressive thoughts [32]. Thus, more research is needed to clarify the relationship between real-life social support, social media social support, and mental health.

#### Problematic social VR use

As mentioned earlier, social VR applications (e.g., VRChat), although exclusively offered in 3-D VR platforms, allow individuals to socially interact with each other and maintain relationships just as traditional 2-D social media does [9]. Additionally, compared to traditional social media on 2-D screens, the 3-dimensional setup of social VR platforms offers more realistic and life-like encounters, capable of mimicking a community where users can maintain relationships and meet new people [33]. The social support received by social VR users may be more similar to that in real life than on 2-D social media, and thus social VR use may contribute to more mental health benefits than traditional social media use. However, when social VR use becomes problematic with addition-like symptoms, it is possible that the negative relationship between problematic social media use and mental health may also be exhibited in the 3-D VR platforms.

Despite the similar social features of social VR and traditional social media, research has rarely investigated how the use of social VR applications is associated with important mental health symptoms such as depression, anxiety, and feelings of isolation. One study linked social VR use to better mental well-being through increased general social support [34]. Another study revealed the benefits of social VR in improving mental health and well-being [35]. However, it remains unknown how problematic social VR use and the amount of social VR use (the type of social VR use that is not necessarily problematic) may work differently with social support and mental health, both compared to one another and compared to traditional 2-D social media use.

**Fig 1:**
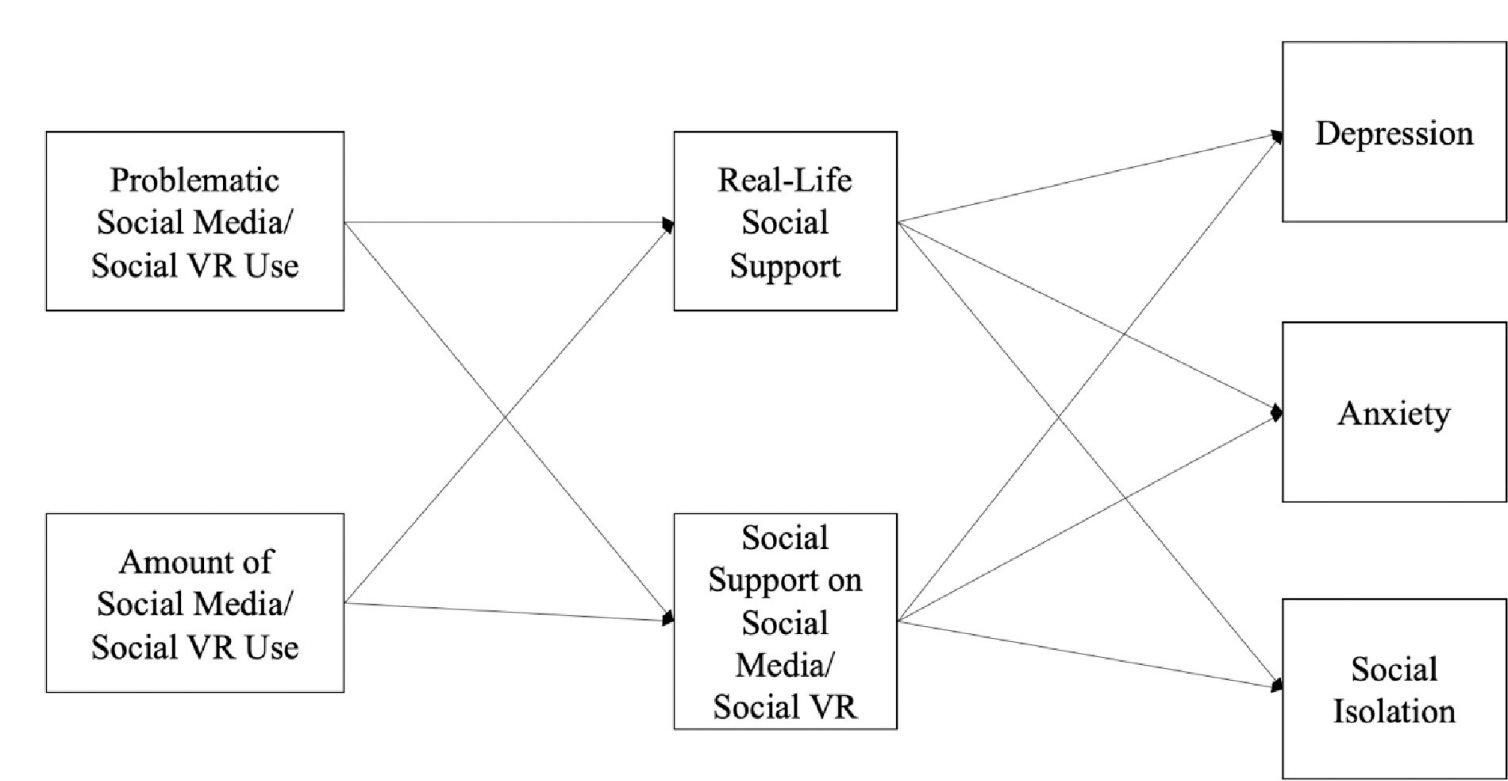
Conceptual model.

Our research, therefore, addresses critical questions regarding the unique association between problematic social media use, general social media use, and mental health. Specifically, we ask: To what extent is mental health associated with problematic social media use and the amount of social media use? How do real-life social support and social media social support mediate this relationship? Finally, are the tested relationships on social media also extant in the context of social VR use? Fig 1 illustrates the conceptual model. Overall, this study extends previous literature in two ways. First, our research is among the first to differentiate the effects of problematic and general social media use on mental health outcomes such as depression, anxiety, and social isolation. Second, by examining these relationships within social VR, we contribute novel insights into the potential mental health implications of this emerging communication technology, grounded in data from active social VR users.

## Study 1

### Study 1 Materials and methods

#### Participants & procedure

An *a priori* power analysis was conducted to calculate the desired sample size to replicate the indirect effects from previous research [23]. Using Monte Carlo samples with 1,000 replications and 20,000 Monte Carlo drawings per replication, a minimum sample of 450 was needed to achieve .80 power with 95% confidence level [36]. Participants were undergraduate students from a large public university in the mid-Atlantic U.S. who completed a 15-minute online survey for course credit. After excluding 73 participants from the analysis due to attention check (i.e., fail to select the choice “often” when instructed to; original n = 521), the final sample was 448 (76% female, 24% male; *M*_age_ = 20.03, *SD*_age_ = 1.36; range_age_ = 18 to 34; racial/ethnic identities: 78% White, 5% Asian, 3% African American, 3% Latino/a, and 12% interracial). The study was approved by the Institutional Review Board of the University of Delaware. Written consent was obtained before participants were allowed to view the rest of the survey. The participant recruitment period of this study was between February 6, 2022, and March 22, 2022. Data associated with Study 1 & 2 are shared on the Open Science Framework. https://osf.io/raq9v/?view_only=58346dc451fb4e019d99cd052e7fa809.

#### Measures

In both studies, all items were randomized within measurement scales. See Tables 1 and 2 for descriptive statistics and correlations.

**Table 1:** Descriptive statistics and correlations (Study 1).

	1	2	3	4	5	6	7
Measurement scale	Very rarely (1)–very often (5)	Zero hour (0)– 11 hours and more (11)	Strongly disagree (-3)–strongly agree (+3)	Strongly disagree (-3)–strongly agree (+3)	Never (1)–always (5)	Never (1)–always (5)	Never (1)–always (5)
Mean	2.67	11.84	2.03	.13	1.94	2.64	2.39
SD	.91	8.79	.92	1.43	.93	.95	.97
Cronbach α	.85	-	.92	.94	.92	.88	.89
1.Problematic Social Media Use	-						
2.Amount of Social Media Use	.25***	-					
3.Real-life Social Support	-.05	.13**	-				
4.Social Support on Social Media	.13**	.14**	.27***	-			
5.Depression	.29***	.04	-.34***	-.06	-		
6.Anxiety	.31***	.07	-.17**	-.03	.63***	-	
7.Social Isolation	.27***	-.02	-.37***	-.14**	.61***	.53***	-

Note. The mean and standard deviation for regular social media use reflects the compiled variable ranging between zero and 77.

*p < .05.

**p < .01.

***p < .001

**Table 2:** Path analysis results (Study 1).

	Coeff.
Problematic social media use→real-life social support	**-.10[-.21, -.01]**
Problematic social media use→social support on social media	.16[-.002, .32]
Amount of social media use→real-life social support	**.01[.01, .02]**
Amount of social media use→social support on social media	**.02[.002, .03]**
Real-life social support→depression	**-.31[-.42, -.21]**
Real-life social support→anxiety	**-.18[-.28, -.07]**
Real-life social support→social isolation	**-.34[-.44, -.23]**
Social support on social media→depression	-.01[-.06, .05]
Social support on social media →anxiety	-.02[-.09, .04]
Social support on social media →social isolation	-.05[-.12, .01]
Problematic social media use→depression	**.27[.19, .36]**
Problematic social media use→anxiety	**.30[.21, .40]**
Problematic social media use→social isolation	**.29[.19, .39]**
Amount of social media use→depression	-.01[-.001, .01]
Amount of social media use→anxiety	-.00[-.01, .01]
Amount of social media use→social isolation	-.004[-.01, .01]
Problematic social media use→real-life social support→depression	**.03[.004, .07]**
Problematic social media use→real-life social support→anxiety	**.02[.003, .05]**
Problematic social media use→real-life social support→social isolation	**.04[.01, .08]**
Problematic social media use→social support on social media→depression	**-.05[-.11, -.002]**
Problematic social media use→social support on social media→ anxiety	**-.03[-.07, -.002]**
Problematic social media use→social support on social media→ social isolation	**-.05[-.12, -.003]**
Amount of social media use→real-life social support→depression	**-.004[-.009, -.002]**
Amount of social media use→real-life social support→ anxiety	**-.003[-.005, -.001]**
Amount of social media use→real-life social support→ social isolation	**-.005[-.01, -.002]**
Amount of social media use→social support on social media→depression	**-.01[-.01, -.001]**
Amount of social media use→social support on social media→ anxiety	**-.003[-.01, -.001]**
Amount of social media use→social support on social media→ social isolation	**-.001[-.01, -.001]**

*Note*: 95% CI is presented in the square bracket next to each coefficient. Coefficients with confidence intervals that do not contain zero are bolded.

R^2^_depression_ = .19. R^2^_anxiety_ = .14. R^2^_social isolation_ = .01

**Problematic social media use** was measured with the 6-item Bergen Social Media Addiction Scale [23, 37]. The **amount of social media use** was captured in two steps. Participants first selected all social media platforms they had used during the past year from 11 most widely used online platforms (e.g., Instagram; [38]). For each selected social media platform, participants reported their average usage on a weekday and a weekend day. An index was then computed by adding the weighted average of weekday social media use (i.e., five weekdays per week) and the weighted average of weekend social media use (i.e., two weekend days per week). **Real-life social support** was measured with the 12-item Multidimensional Scale of Perceived Social Support (MSPMS; [39]). **Social media social support** was measured with a modified version of MSPMS. When relatable, the phrase “on social media” was added to the statement (e.g., “There is a special person who is around on social media when I am in need.”). **Depression** was measured with the 4-item short form of the PROMIS depression scale [40]. **Anxiety** was measured with the 4-item short form of the PROMIS depression scale [40]. **Social Isolation** was measured the 4-item short form of the PROMIS social isolation scale [23, 41]. For depression, anxiety, and social isolation, participants rated how frequently they felt for each item (e.g., “I felt depressed.”) in the past 7 days.

#### Statistical analysis

Analysis in both studies was performed using the Lavaan package [42] in R (version 4.3.1). Because we were interested in testing the direct and indirect relationships between variables, a saturated model was tested and thus the model indices were not meaningful for interpretation. Error terms were allowed to covary within each model stage (e.g., mediators with mediators, outcomes with outcomes). Participants’ age, race, and biological sex were analyzed as covariates. Unstandardized coefficients were reported using 5,000 bias-corrected bootstrapped samples.

### Study 1 Results and discussion

In Study 1 we tested the mediating relationships of traditional 2-D social media use (problematic and the amount of), real-life and online social support, and mental health symptoms (i.e., depression, anxiety, social isolation). See Table 1 for descriptive statistics and Table 2 for the path analysis results. With direct effects, increased problematic social media use was significantly associated with increased social media social support but nonsignificantly associated with real-life social support. However, increased amount of social media use was associated with increased real-life social support and social media social support. Real-life social support was negatively associated with depression, anxiety, and social isolation, whereas problematic social media use was positively associated with all three. Relationships between social media social support and mental health were nonsignficant. Relationships between the amount of social media use and mental health were also nonsignificant.

For indirect effects with problematic social media use as the exogenous variable, increased problematic social media use was associated with decreased real-life social support, which further impaired mental health. However, increased problematic social media use was associated with greater perceived social media social support, which bolstered mental health. For indirect effects with the amount of social media use as the exogenous variable, all paths through real-life social support and social media social support were statistically significant. The amount of social media use was associated with increased real-life social support and social media social support, which were both associated with lower depression, anxiety, and social isolation.

Problematic social media use was associated with weaker real-life social support, replicating previous findings [23]. In addition, problematic social media use was found to be nonsignificantly related to social media social support. However, when parsing out the influence of problematic social media use, the amount of social media use positively contributed to real-life social support and social media social support. Problematic social media use was also related to poorer mental health, whereas non-problematic social media use (the amount of social media use) was related to better mental health. Overall, these findings revealed the divergent roles of problematic social media use and the amount of social media use on mental health-related issues: problematic social media use may potentially impair mental health, but the amount of social media use may benefit users’ mental health.

These results also echo the findings from previous research that demonstrated the negative effects of problematic social media use on mental health through reduced perceived social support from everyday life [22, 31]. With social media social support as the mediator, both problematic social media use and the amount of social media use contributed to better mental health. Notably, this finding contradicts a previous study where only real-life social support but *not* social media social support mediated between problematic social media use and mental health [23]. However, Meshi and Ellithorpe did not include the amount of social media use in their model, thus it is possible that the nonsignificant relationship between problematic social media use, social media social support, and mental health in their study was due to the amount of social media use as a confounder. This finding demonstrated that problematic social media use may have an ambivalent effect on mental health, rather than a completely negative effect. This finding further implies the importance of capturing problematic and non-problematic social media use simultaneously when studying the relationship between social media use and mental health.

## Study 2

In Study 2, we extended our inquiry to an emerging communication technology, social VR, to explore whether the relationships identified between traditional social media use and mental health would persist within this novel context. Characterized by unique communication modalities, social VR employs embodiment through avatars and immersive experiences via head-mounted displays, and provides affordances for synchronous verbal and non-verbal communication [43]. These unique aspects of social VR interactions may shape user perceptions and experiences of social support in different ways than traditional social media, subsequently influencing their mental well-being [44].

In line with the approach of Study 1, we tested a similar model, this time incorporating problematic social VR use and the amount of social VR use as the exogenous variables (Fig 1). Given the nascent stage of social VR’s prevalence compared to traditional social media, we specifically recruited active social VR users as study participants.

### Study 2 Materials and methods

#### Participants & procedure

Social VR users who were aged 18 or older were recruited through social VR and VR-related forums and groups on Reddit and Facebook [34]. Participants used the link in the post to participate in the 12-minute online survey and were allowed to share the survey link with others. Three participants who provided contact information were randomly selected to win one of the three $100 Amazon gift cards. The study was approved by the Institutional Review Board of Georgia State University. Written consent was obtained before participants were allowed to view the rest of the survey. The participant recruitment period of this study was from March 3, 2023, to April 20, 2023.

Due to the novelty of the current context and the similarity between Studies 1 & 2, we used the same power analysis result (desired n = 450) from Study 1 for this study. Of the initial 1103 participants, 135 were excluded from analysis due to duplicate responses from multiple platforms. Additional 493 participants were excluded from analysis due to attention check (i.e., failed to select “often” when instructed to), impossible age (e.g., older than 150), or duplicate IP addresses. Finally, 11 participants who reported zero hours of weekly social VR usage during the past year were excluded. The final sample (n = 464) had an age range of 18 to 68 (*M* = 29.88; *SD* = 7.36), comprised of 40% women, 57% men, and 3% non-binary/other (racial breakdown: 85% White, 6% interracial, 4% Black, 2% Hispanic or Latino/a, 3% other identities).

#### Measures

**Problematic social VR use** was identical as problematic social media use from Study 1 except that “social media” was replaced by “social VR” when applicable, where participants were instructed to think about their “interactions on social VR platforms (e.g., Rec Room, VRChat, Horizon Worlds)”. For **the amount of social VR use**, participants first selected all platforms they had used during the past year from a pool of 12 popular social VR platforms. The 12 popular SVR platforms in the survey include Horizon Worlds, Horizon Venues, Bigscreen, Rec Room, AlcoveVR, Half + Half, VRChat, AltspaceVR, vTimeXR, BeanVR, Couch, Sansar. An “other” option allowed for unlisted platforms. Then, for each selected social VR platform, participants reported their average usage on a weekday and a weekend day. The index was the sum of the weighted average weekday social VR use and weighted weekend social VR use. **Social Support on Social VR** measure was identical as the social media social support measure from Study 1 except that “social media” was replaced by “social VR” when applicable. Measures of real-life social support, depression, anxiety, and social isolation were identical to Study 1.

#### Statistical analysis

The model-building procedure was the same as Study 1. Participant age, gender, and racial identity were analyzed as covariates.

### Study 2 Results and discussion

In Study 2 we tested the same mediating relationships in Study 1 in a different context, namely social VR. Particularly, we investigated how real-life social support and social VR social support would mediate the relationships between active users’ problematic and the amount of social VR use and their mental health. See Table 3 for descriptive statistics and Table 4 for the path analysis results. With direct effects, real-life social support was significantly and negatively predicted by problematic social VR use but not the amount of social VR use. Social VR social support was significantly and positively predicted by the amount of social VR use but not by problematic social VR use.

**Table 3:** Descriptive statistics and correlations (Study 2).

	1	2	3	4	5	6	7
Measurement scale	Very rarely (1)–very often (5)	Zero hour (0)– 11 hours and more (11)	Strongly disagree (-3)–strongly agree (+3)	Strongly disagree (-3)–strongly agree (+3)	Never (1)–always (5)	Never (1)–always (5)	Never (1)–always (5)
Mean	1.85	4.31	5.56	4.96	1.80	1.75	1.76
SD	.67	3.64	.95	1.11	.69	.75	.85
Cronbach α	.82	-	.94	.96	.80	.83	.87
1.Problematic Social VR Use	-						
2. Amount of Social VR Use	.14***	-					
3.Real-life Social Support	-.45***	-.03	-				
4.Social Support on Social VR	-.16***	.18***	.48***	-			
5.Depression	.61***	.06	-.54***	-.39***	-		
6.Anxiety	.64***	-.04	-.53***	-.53***	.77***	-	
7.Social Isolation	.68***	.01	-.65***	-.48***	.77***	.79***	-

Note. The mean and standard deviation for regular social VR use reflects the compiled variable ranging between zero and 77.

***p < .001

**Table 4:** Path analysis results (Study 2).

	Coeff.
Problematic social VR use→real-life social support	**-.62[-.80, -.44]**
Problematic social VR use→social support on social VR	-.06[-.25, .14]
Amount of social VR use→real-life social support	-.02[-.05, .04]
Amount of social VR use→social support on social VR	**.06[.04, .15]**
Real-life social support→depression	**-.16[-.29, -.06]**
Real-life social support→anxiety	**-.11[-.24, -.01]**
Real-life social support→social isolation	**-.27[-.40, -.17]**
Social support on social VR →depression	**-.13[-.20, -.06]**
Social support on social VR →anxiety	**-.19 [-.26, -.12]**
Social support on social VR →social isolation	**-.17[-.25, -.10]**
Problematic social VR use→depression	**.50[.36, .63]**
Problematic social VR use→anxiety	**.59[.47, .72]**
Problematic social VR use→social isolation	**.65[.50, .78]**
Amount of social VR use→depression	-.005[-.01, .05]
Amount of social VR use→anxiety	-.01[-.03, .02]
Amount of social VR use→social isolation	-.01[-.03, .02]
Problematic social VR use→real-life social support→depression	**.10[.03, .20]**
Problematic social VR use→real-life social support→anxiety	**.07[.005, .16]**
Problematic social VR use→real-life social support→social isolation	**.17[.09, .28]**
Problematic social VR use→social support on social VR →depression	.01[-.02, .04]
Problematic social VR use→social support on social VR → anxiety	.01[-.03, .05]
Problematic social VR use→social support on social VR → social isolation	.01[-.02, .05]
Amount of social VR use→real-life social support→depression	-.003[-.01, .01]
Amount of social VR use→real-life social support→ anxiety	-.005[-.007, .004]
Amount of social VR use→real-life social support→ social isolation	-.005[-.01, .01]
Amount of social VR use→social support on social VR →depression	**-.01[-.02, -.003]**
Amount of social VR use→social support on social VR → anxiety	**-.01[-.03, -.01]**
Amount of social VR use→social support on social VR → social isolation	**-.01[-.03, -.004]**

*Note*: 95% CI is presented in the square bracket next to each coefficient. Coefficients with confidence intervals that do not contain zero are bolded.

R^2^_depression_ = .63. R^2^_anxiety_ = .62. R^2^_social isolation_ = .68.

Increased real-life social support was associated with decreased depression, anxiety, and social isolation. Increased social VR social support was associated with decreased depression, anxiety, and social isolation. Problematic social VR use was associated with increased depression, anxiety, and social isolation, whereas the amount of social VR use was not significantly associated with any mental health measure.

With indirect effects, increased problematic social VR use was associated with decreased real-life social support, which contributed to greater depression, anxiety, and social isolation. All paths between the amount of social VR use, real-life social support, and mental health were nonsignificant. Increased amount of social VR use was associated with increased social VR social support, which was associated with lower depression, anxiety, and social isolation. All paths between problematic social VR use, social VR social support, and mental health issues were nonsignificant.

Problematic social VR use was associated with weaker real-life social support, aligning with prior findings with problematic use of traditional social media [22]. However, problematic social VR use was not related to social VR social support in the tested model; instead, social VR social support was positively predicted by the amount of social VR use. These findings suggest that problematic social VR use, similar to problematic use of traditional social media, might divert users from real-life social interactions, weakening perceived real-life social support; instead, the amount of social VR use positively predicted social VR social support, implying a positive influence on user perceptions of support within the virtual environment.

Notably, depression, anxiety, and social isolation were significantly predicted by problematic social VR use but not the amount of social VR use, which is consistent with findings from Study 1. Inconsistent with Study 1, both real-life and social VR social support were found to improve mental health. This implies social VR, when used in a non-problematic manner, may offer a more supportive environment than traditional social media for users to experience support from other users, thereby yielding desirable mental health outcomes akin to those seen with real-world social support. Further research is encouraged to replicate and better understand these relationships.

The indirect effects between problematic social VR use, social VR social support, and mental health issues largely align with research on traditional social media [23]. The amount of social VR use, though, was associated with reduced depression, anxiety, and social isolation only through greater social VR but *not* real-life social support. Prior research on social support and mental health in the context of traditional social media tends to find stronger effects from real-life social support than social media social support [23, 30], which contradicts the current finding with social VR. This shift from real-life social support to social VR social support could be attributed to the immersive, interactive, and embodied experiences with virtual reality [45] which allow users to engage in more immediate and naturalistic social interactions. When utilized properly, these immersive social interactions may help users build more engaging relationships compared to text-based and asynchronous interactions via social media. This interpretation could explain why the influence of social VR social support on mental health is more pronounced compared to real-life social support in the context of social VR use. Thus, these findings suggest the potential of social VR as a meaningful platform for building supportive social connections and promoting mental well-being.

## General discussion

This study provides empirical insights with societal benefits. By distinguishing problematic from the general use of social media and social VR, our findings clarify potential mental health risks and benefits associated with these platforms, informing targeted interventions and healthy usage strategies.

The results of the two studies suggest that problematic social media use and the amount of social media use (or, problematic social VR use and the amount of social VR use) may work differently in processes related to mental health as well as perceived social support. In both studies, only problematic social media use (or problematic social VR use) but not the amount of social media use (or the amount of social VR use) was directly related to depression, anxiety, and isolation. These results provide insight into the inconsistent findings regarding the social media use-mental health relationship in previous research. When using the duration of social media use (e.g., in hours) as a predictor, some studies have found that increased social media use is related to worse mental health [13], while others have found no such relationship [16]. With our results we encourage future research to consider the possible separate roles of problematic social media use and the amount of social media use in users’ mental health. Another future research direction is to test the nonlinear relationship between social media use and mental health. Indeed, a recent systematic review concluded that the relationship between the time spent on social media and mental health issues may not be linear [12]. More research is needed to further understand this relationship including boundary conditions, the threshold between problematic and non-problematic social media use, and the effect of time.

Study 2 demonstrated that problematic social VR use was related to decreased real-life but not social VR social support, which consequently mitigated mental health issues. This result aligns with previous research on problematic social media use, where real-life social support was more effective in combatting mental health issues than social media social support [23]. However, the amount of social VR use was associated with enhanced social VR social support, but not real-life social support, leading to better mental health outcomes. This finding, which contrasts with Study 1’s findings, emphasizes the potential impact of the immersive 3D virtual environments on perceived social support. Future research should further explore how the technological affordance of immersion may contribute to mediated social support as well as improving mental well-being.

In sum, our findings underscore how differentiating problematic from non-problematic use of social media and social VR can guide interventions targeting mental health risks. The unique benefits of social VR suggest that, when used positively, immersive digital interactions could serve as supportive, engaging communities that counteract mental health challenges. These findings are especially timely as social VR attracts more users, presenting an opportunity for platform designers, mental health advocates, and policymakers to shape environments that enhance connectedness and mitigate mental health risks.

Our research has limitations. First, both studies had nonrepresentative samples. Although our findings largely replicated previous research, the nonrepresentative nature of the sample limits our results’ generalizability. Additionally, mediation analysis was conducted with cross-sectional data, therefore we caution readers to avoid drawing causal conclusions from our results. It is likely that the relationships between social media and VR use with mental health are dynamic and mutually reinforcing over time. We thus recommend future research utilize experimental and longitudinal methods to test the same mechanisms to cross-validate the current results. Lastly, it is recommended that future research use both qualitative and quantitative approaches to study the focal phenomenon with more depth and to better understand idiosyncratic context.

## Conclusion

In conclusion, our results reveal the importance of understanding the potentially divergent roles of problematic social media use and the amount of social media use on users’ mental health. In Study 2 we also found that the amount of social VR use was associated with more perceived social support on social VR than in real-life which contradicts previous findings in traditional social media. This novel finding should encourage VR researchers, VR developers, and policymakers to further explore how social VR may promote social support as well as mental well-being.
